# Dietary supplementation with omega-3 fatty acid attenuates 5-fluorouracil induced mucositis in mice

**DOI:** 10.1186/s12944-015-0052-z

**Published:** 2015-06-12

**Authors:** Simone de Vasconcelos Generoso, Núbia Morais Rodrigues, Luísa Martins Trindade, Nivea Carolina Paiva, Valbert Nascimento Cardoso, Cláudia Martins Carneiro, Adaliene Versiani de Matos Ferreira, Ana Maria Caetano Faria, Tatiani Uceli Maioli

**Affiliations:** Departamento de Nutrição, Escola de Enfermagem, Universidade Federal de Minas Gerais, Belo Horizonte, Brazil; Núcleo de Pesquisa em Ciências Biológicas, Instituto de Ciências Exatas e Biológicas, Universidade Federal de Ouro Preto, Ouro Preto, Brazil; Departamento de Análises Clínicas e Toxicológicas, Escola de Farmácia, Universidade Federal de Minas Gerais, Belo Horizonte, Brazil; Departamento de Bioquímica e Imunologia, Instituto de Ciências Biológicas, Universidade Federal de Minas Gerais, Belo Horizonte, Brazil

**Keywords:** Omega-3 fatty acid, Mucositis, Intestinal damage, ácido graxo ômega-3, mucosite, dano intestinal

## Abstract

**Background:**

Studies showed the positive effects of omega-3 fatty acid (n-3 FA) for the treatment of inflammatory bowel disease as it alleviated the symptoms and promoted better mucosal integrity. The objective of this study was to determine whether a diet with the addition of n-3 FA helps control the inflammation observed in 5-fluorouracil (5-FU) induced mucositis.

**Methods:**

BALB/c mice were randomly divided into four groups as follows: 1: control (CTL), fed a standard chow diet; 2: CTL + n-3 FA – n-3 FA, fed a diet with n-3; 3: mucositis (MUC), fed a standard chow diet and subjected to mucositis; and 4: MUC+ n-3 FA, fed a diet with n-3 FA and subjected to mucositis. On the 8^th^ day, the animals of the MUC and MUC + n-3 FA groups received an intraperitoneal injection of 300 mg/kg 5-FU for mucositis induction. After 24 h or 72 h, all mice were euthanized and evaluated for intestinal permeability, bacterial translocation, intestinal histology and apoptosis.

**Results:**

Mice that received the diet with n-3 FA and a 5-FU injection showed less weight loss compared to the animals of the MUC group (*p* < 0.005). Decreased intestinal permeability and bacterial translocation were also observed in animals fed n-3 FA, and these mice underwent mucositis compared to the MUC group (*p* < 0.005). These data were associated with mucosal integrity and a reduced number of apoptotic cells in the ileum mucosa compared to the mice that received the control diet and 5-FU injection.

**Conclusion:**

Together, these results show that omega-3 fatty acid decreases the mucosal damage caused by 5-FU-induced mucositis.

## Resumo

**Introdução:**

Estudos têm demonstrado efeitos positivos da utilização do ácido graxo ômega-3 (n-3 AG) no tratamento de doenças inflamatórias do intestino, aliviando os sintomas e promovendo melhora da integridade da mucosa. Assim, no presente estudo, foi avaliado o potencial de uma dieta adicionada n-3 AG poderia ajudar a controlar a inflamação observada na mucosite intestinal induzida pelo 5-fluoracil (5-FU).

**Métodos:**

Camundongos BALB/c foram divididos aleatoriamente em quatro grupos: 1. Controle (CTL) - alimentados com ração padrão; 2. CTL + n-3 AG - alimentados com uma dieta adicionada de n-3 AG; 3. Mucosite (MUC) - alimentados com a dieta ração padrão e submetidos à indução da mucosite; 4. MUC + n-3 FA – alimentados com dieta adicionada de n-3 AG e submetidos à indução da mucosite. No oitavo dia, os animais dos grupos MUC e MUC + n-3 AG receberam uma injeção intraperitoneal de 300 mg/kg de 5-FU para a indução da mucosite. Após 24 h ou 72 h, todos os camundongos foram eutanaziados para a avaliação da permeabilidade intestinal, translocação bacteriana, histologia intestinal e ensaio de apoptose.

**Resultados:**

Os animais que receberam a dieta adicionada de n-3 AG e injeção de 5-FU mostraram menor perda de peso comparado com os animais do grupo MUC (*p* <0,005). Foi observado diminuição da permeabilidade intestinal e translocação bacteriana nos animais alimentados com n-3 AG e submetidos a mucosite. Estes dados foram associadas com melhor integridade da mucosa e uma redução do número de células apoptóticas na mucosa do íleo em comparação com os camundongos que receberam ração controle dieta e injeção com 5-FU.

**Conclusão:**

Estes resultados mostram que o ácido graxo ômega-3 pode diminuir o dano da mucosa causada pela mucosite induzida pelo 5-FU.

## Background

Polyunsaturated fatty acids (PUFA) are a family of lipids with two or more double bonds [1]. The primary PUFAs are linoleic acid (C18:2n-6) and alpha-linolenic acid (C18:3n-3), and both are essential for metabolism in mammals [1, 2]. PUFAs are important components of cell membranes because of their fluidity [3, 4]. These molecules are substrates for inflammatory and anti-inflammatory eicosanoid production, as exemplified by prostaglandins (PG) and leukotrienes (LTB) [1–3, 5]. In mammalian cells, omega-6 (n − 6) fatty acid (FA) and omega-3 (n − 3) fatty acid (FA) compete for metabolism by the same enzyme, yielding arachidonic acid or eicosapentaenoic acid (EPA) and docosahexaenoic acid (DHA), respectively. EPA and DHA can replace arachidonic acid in cell membranes and suppress pro-inflammatory mediator production [4].

Current studies have shown the positive effects of n-3 FA for the treatment of inflammatory bowel disease (IBD), alleviating the symptoms and promoting better mucosal integrity [4, 6, 7]. The most likely mechanism involved is the decreased intestinal production of PG and LTB of odd series [7] precursors of pro-inflammatory cytokines [4, 6]. Additionally, n-3 FA decreased the protein expression of intestinal NFκB p65 related to apoptotic cells [8]. Based on this evidence, supplementation with omega-3 fatty acid may be a good alternative for treating damage caused by mucositis.

Mucositis is the mucosal damage that is secondary to chemotherapy or radiotherapy [9], affecting up to 60 % of patients receiving high-dose chemotherapy and almost 100 % of patients undergoing preconditioning chemotherapy regimens for stem cell transplant [10, 11]. It is characterized by inflammation and/or gastrointestinal tract ulcers, leading to gut mucosal dysfunction, such as diarrhea, weight loss and increased intestinal permeability, which can lead to infections and potentially fatal consequences [12, 13].

This phenomenon occurs because anti-metabolite agents, such as 5-fluorouracil (5-FU), exert their functions in cells that are in the S phase of the cell cycle, inhibiting the synthesis of essential components of deoxyribonucleic acid (DNA) and ribonucleic acid (RNA), interrupting cell proliferation [14, 15]. Consequently, there is a promotion of oxidative stress leading to cytotoxic effects on the cells. However, these effects are not restricted to cancer cells. This drug also acts on all proliferative cells, such as mucosal membrane cells; therefore, they are equally sensitive to damage [14].

A previous study from our research group showed that mice that underwent mucositis induced by 5-FU showed a decrease in food consumption, greater weight loss, and increased intestinal permeability and inflammation [16]. Therefore, it is important to investigate the role of n-3 FA in the intestinal damage induced by 5-FU.

## Results

### Diet with omega-3 fatty acid prevents weight loss, intestinal mucosal damage and bacteria translocation

**Fig. 1:**
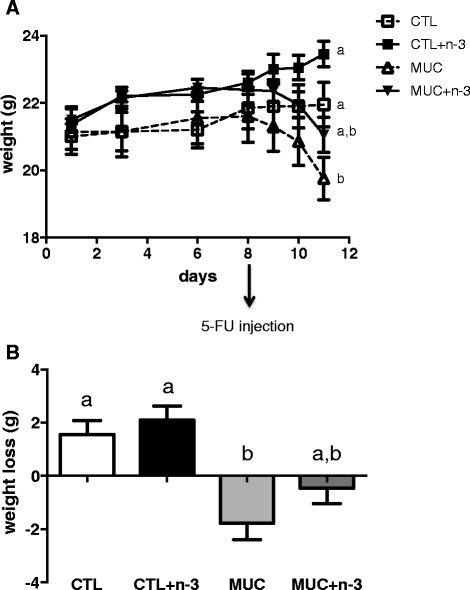
Weight progress. **a** The mice weights were monitored from the first experimental day to the 11th day. On 8th day mice received an intraperitoneal injection of 5-FU. **b** The weight loss delta was performed 72 h after mucositis induction subtracting the weight from the first experimental day to the last weight measured. Different letters indicate statistical significance at 11th day (*p* < 0.05), n = 10

Figure 1a shows that after both 24 and 72 h of mucositis induction, mice from the MUC group had higher weight loss compared with the control group (*p* < 0.05). However, mice a fed diet with n-3 FA that underwent mucositis had reduced weight loss (*p* < 0.05) (Fig. 1a). The difference between the weight on the first day and 72 h after the induction of mucositis showed that mice fed a diet with n-3 FA presented smaller weight loss than the mice of the MUC group (Fig. 1b) (*p* < 0.05).

**Fig. 2:**
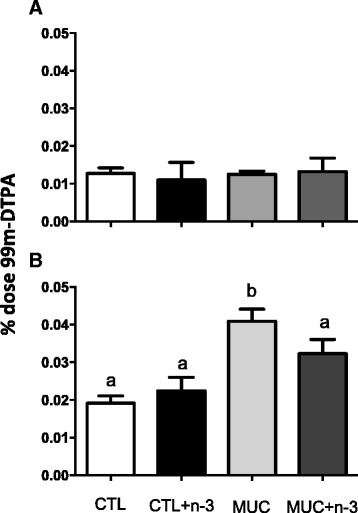
The effect of omega-3 fatty acid on intestinal permeability. **a** Intestinal permeability 24 h after of mucositis induction. **b** Intestinal permeability 72 h after mucositis induction. Different letters indicate statistical significance, measured by ANOVA (*p* < 0.05). Data are representative of three different experiments with 5 mice/group each

Another consequence of mucositis is intestinal mucosa damage. To determine whether n-3 FA prevents mucosal damage and its initiation, intestinal permeability was evaluated at two time points, 24 h and 72 h. Intestinal permeability was not altered 24 h after mucositis induction (Fig. 2a). However, 72 h after the 5-FU injection (Fig. 2b), the intestinal permeability was higher in the MUC group compared to the CTL group (*p* < 0.05), whereas, the animals that received n-3 FA and underwent mucositis had decreased intestinal permeability compared to the MUC group mice (*p* < 0.05).

Bacteria translocation was performed 72 h after mucositis induction (Table 1) and was decreased in mice fed a diet with n-3 FA. The amount of 99mTc-*E. coli* was lower in the lungs, livers, mesenteric lymph nodes and spleens of the MUC+ n-3 FA mice than in the MUC mice (*p* < 0.05), indicating that n-3 FA prevented the disruption of the intestinal barrier and, consequently, decreased the BT levels.

**Table 1:** Bacterial translocation – Tc^99m^ -E. coli

Group/Tissue	CTL	CLT + n-3 FA	MUC	MUC+ n-3 FA
*Blood*	23.449,19^a^	21.678,22^a^	19.376,46^a^	15.862,66^a^
*Liver*	23.078,86^a^	15.669,89^a^	125.713,20^b^	49.781,72^a^
*Spleen*	23.840,66^a^	62.647,74^a^	189.238,16^b^	70.816,87^a^
*MLN*	56.674,92^a^	48.248,80^a^	236.878,69^b^	156.677,79^a^

The data are expressed in cpm/g of tissue. Letters a and b = *p* < 0,05 in the same tissue. MLN: mesenteric lymph node

**Fig. 3:**
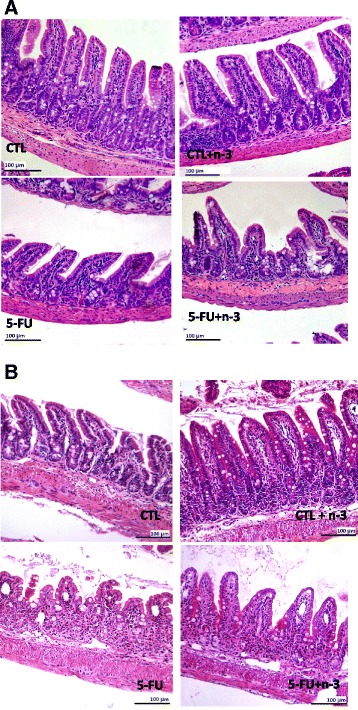
Ingestion of omega-3 fatty acid inhibited ileum mucosa damage caused by 5-FU. **a** Normal histological aspects in the ileum mucosa were observed 24 h after 5-FU injection in all groups. **b** Increased cell infiltration in the lamina propria was observed in the mice that developed mucositis and mucosal architecture disruption was also observed 72 h after 5-FU injection. Bar = 100 μm. The slices were stained with H&E. 100 ×

**Fig. 4:**
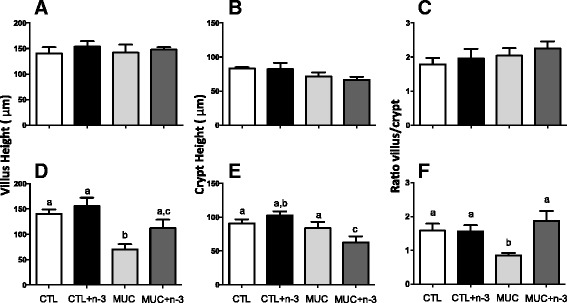
Omega-3 fatty acid is able to maintain ileum mucosal architecture in mice with mucositis. **a**, **b** and **c**) Morphometrical analyses of the small intestine slices from the mice treated or not with the n-3-rich diet were performed using ImageJ software 24 h after mucosittis induction. **d**, **e** and **f** Morphomeric analyses after mucositis induction. **a** and **b** Villus mean height (μm), (**b** and **e**) Lieberkün crypt mean height (μm) and (**c** and **e**) ratio between villus and crypt mean heights. Different letters indicate statistical significance calculated by ANOVA (*p* < 0.05), n = 5

Histology analyses were used to assess alterations in the ileum mucosa. Sections of small intestine were obtained and analyzed 24 h and 72 h after 5-FU injection. Figure 3a shows that at 24 h after 5-FU injection, no alterations occurred in the ileum mucosa. Although 72 h after mucositis induction, mice from the MUC group showed lesions in the small intestine with cell infiltration in the lamina propria, as well as inflammation in the submucosa and muscular layers. Mice fed a diet with n-3 FA that underwent mucositis showed more preserved ileum mucosa that the MUC group mice and also showed a similar histology compared to the mice that did not received 5-FU (Fig. 3b). The morphometric analyses showed decreased villus and increased crypt heights (Fig. 4d and f) in the MUC group, whereas in the MUC+ n-3FA group, the villus (Fig. 4d) and crypt (Fig. 4e) heights were similar to the CTL and CTL+ n-3FA groups (*p* < 0.05). Additionally, the mice that received the diet with n-3 FA showed reversed parameters and normal villus/crypt ratios (Fig. 4f). No alterations in the morphometric analyses were observed 24 h after 5-FU injection (Fig. 4a, b and c).

### Diet with omega-3 fatty acid can prevent apoptosis induced by 5-FU in the intestinal mucosa

**Fig. 5:**
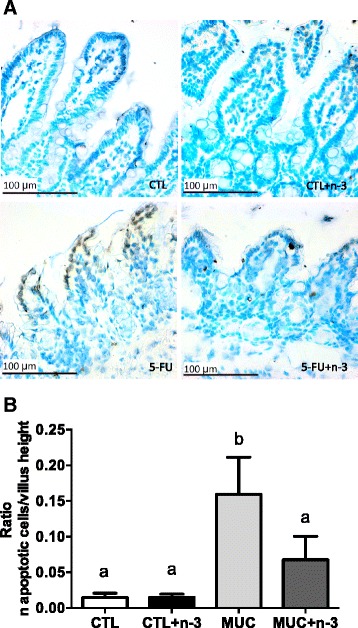
Omega-3 fatty acid prevents apoptosis in the ileum mucosa after 5-FU injection. Ileum fragments were subjected to TUNEL labeling and morphometric analyses. **a** Few apoptotic cells were labeled in the mucosa from the CTL, CTL + n-3 FA and MUC+ n-3 FA mice. Increased apoptotic cells were observed in the mice that developed mucositis (MUC). Bar = 100 μm. **b** Villus height and apoptotic cell number ratio. Different letters indicate statistical significance (*p* < 0.05) that were measured by ANOVA (n = 5/group)

To determine the mechanism by which n-3 FA prevents intestinal damage, mucosal cell apoptosis was measured by immunohistochemistry using TUNEL. Figure 5a shows the labeling of epithelial cells from MUC mice. However, MUC+ n-3FA mice showed results similar to the CTL or CTL+ n-3FA group mice. Apoptotic cell quantification in the ileum mucosa showed a reduced number of apoptotic cells in the MUC+ n-3FA group mucosa compared to the MUC group (Fig. 5b). Therefore, these data suggest that a diet with n-3 FA prevents intestinal cell apoptosis.

## Discussion

In the current study, we demonstrated the positive effect of omega-3 fatty acid in an experimental model of mucositis induced by 5-FU. Our results showed decreased weight loss and intestinal permeability with controlled bacterial translocation and mucosal integrity maintenance in animals fed a diet with n-3 FA 72 h after mucositis induction via the inhibition of apoptosis in ileum mucosal cells.

Analyses of the fatty acid profiles of a diet consisting of fish oil had higher EPA and DHA concentrations compared to the control diet. Although expected, this assessment was important because the n-3 FA used is a commercial product and the quantities expressed on the label may be different. Furthermore, the dietary intake of pre formed EPA and DHA may be more effective than only alpha-linoleic acid consumption [17]. There is convincing evidence that the effects of n-3 FA supplementation are due to the beneficial properties of EPA and DHA [18, 19]. Both molecules have long been proposed to improve health by controlling blood pressure, alleviating symptoms of rheumatoid arthritis and depression, as well as attenuating the progression of Alzheimer’s disease and gut inflammation [16, 19–21]. Therefore, we used n-3 to determine whether it controls inflammation in mucositis.

Mucositis is a chemotherapy side effect that is characterized by inflammation from mouth to anus. In humans, it is characterized by weight loss, generalized infection and longer hospitalization time [12, 13]. In mice, the inflammation site depends on the mouse strain and the general effects of the disease [21, 22]. In a previous study with BALB/c mice, gut inflammation was observed mainly in the terminal jejunum and ileum, with significant weight loss [23, 24]. This current study showed high weight loss in animals of the MUC group, 24 h and 72 h post 5-FU injection. However, when mice were fed a diet with n-3 FA and underwent mucositis, they demonstrate less weight loss, at both time points analyzed. These results are consistent with those of Koppelmann et al. [22], who observed less weight loss in rats supplemented with n-3 FA and subjected to methotrexate (MTX)-induced intestinal damage compared to the control group [25]. These results confirm that n-3 diet can prevent weight loss during chemotherapy.

The weight loss observed in animals with mucositis can be explained by the lower absorption of nutrients due to the destruction of the intestinal mucosa architecture with ulcerations and increased intestinal permeability [23, 26]. These alterations allow the luminal contents, including pathogens and toxins, to pass through the intestinal epithelial cell layer, leading to bacterial translocation (BT), which is a cause of several inflammatory reactions observed in the mucositis model [27].

In this study, intestinal permeability was evaluated by measuring blood radioactivity after the oral intake of 99mTc-DTPA. This compound is a disodium complex with a molecular weight of 549 Da and a half-life of 6 h, which satisfies the criteria for a marker that can measure intestinal permeability. Our results showed increased intestinal permeability 72 h after 5-FU injection in animals of the MUC group compared to the animals in the CTL group, but this effect was not observed 24 h after mucositis induction. However, mice that received a diet with n-3 (MUC + n-3) did not have increased intestinal permeability, and this may be the reason for the prevention of weight loss is this group.

Because we did not observe any difference in intestinal permeability at 24 h, we performed the bacteria translocation assay only after 72 h. Greater 99mTc-*E. coli* uptake was observed in the liver, spleen and mesenteric lymph nodes from animals subjected to mucositis compared to the CTL group. This is related to the increased intestinal permeability [28], which allows the passage of microorganisms into the intestinal lumen, leading to sepsis and death [29]. In this study, mice fed a diet with n-3FA had decreased intestinal permeability and bacterial translocation, even after mucositis induction, compared to mice that did not receive n-3 FA. Bacterial translocation is possible when inflammation and lesions on the small and large intestine are present [28, 30, 31]. One possible explanation for this protective effect of n-3 FA is its regulatory functional capacity to control inflammation, leading to a reduction in mucosal damage [17, 25, 32, 33]. Another explanation for the protective effects of n-3 FA is its action on intestinal cell junctions, such as tight junctions, occludin and ZO-1, which are responsible for regulating paracellular permeability [34, 35]. Beguin and colleagues, [34], using an in vitro intestinal cell translocation model cultivated with omega-3 fatty acid and exposed to inflammatory stimuli, showed that DHA prevented the redistribution of occludin and ZO-1 and that this is induced by inflammatory cytokines. These effects were also observed by Li et al., 2014 and [35].

Weight loss and increased intestinal permeability following 5-FU-induced mucositis are directly associated with intestinal mucosa damage [16, 20, 21]. To determine whether n-3 treatment controls inflammation in the present mucositis model, histological analyses were also performed to determine whether the permeability and BT control occurred via maintenance of the intestinal mucosa architecture. The histological analyses 24 h after 5-FU injection showed no alteration in the ileum mucosa of the small intestine. However 72 h after 5-FU injection, we observed a disruption of the ileum mucosa with increased cellular infiltration in the lamina propria and a decreased villus/crypt ratio. However, mice injected with 5-FU and fed a diet with n-3 showed decreased mucosal inflammation with maintenance of the villus and crypt length, making the MUC + n-3 mice similar to the CTL mice. It was previously described that n-3 FA consumption significantly attenuates intestinal injury [22, 36].

To assess the mechanism by which n-3 FA prevents intestinal damage, we performed an apoptosis assay in the mice ileum mucosa 72 h after the 5-FU injection. It is known that associated mechanisms related to 5-FU-induced mucositis are complex, involving DNA damage, RNA transcription impairment and subsequent apoptosis [37]. Increased apoptosis occurs due to DNA condensation and the activation of caspases with decreased proliferation of villus cells [38, 39]. By contrast, increased EPA or DHA concentrations in the membrane of cells reduce caspase 9 and 3 activation and decrease cytochrome c release, which regulates cellular oxidative stress and controls endothelial cell dysfunction [36, 40]. Using a TUNEL assay, we observed a decreased rate of apoptotic cells in the ileum mucosa of n-3 treated mice that underwent mucositis compared to mice that did not receive n-3 FA. The most likely mechanism is the decrease of caspase activation promoted by n-3 FA in the cells from the ileum mucosa [41, 42]. In the present study, n-3 prevented mucosal apoptosis, and this effect may be related to the mucosal integrity and decreased weight loss in treated mice. However, additional studies should be performed to identify the molecules that are suppressed by n-3 in the apoptosis pathway.

Other nutritional or pharmaceutical agents are also available in a mucositis model. The results changed according to the type or concentration of agent or substance used. Some studies showed that amino acids, such as arginine, glutamine and citrulline, can promote partial mucosal recovery after mucositis induction [43–45]. Probiotics and prebiotics have also been extensively studied in mucositis, but with controversial results [16, 46–48]. A recent study showed that an herbal preparation with antioxidant properties can decrease the severity of radiation-induced mucositis [49]. Fish oil has been used as an agent to control intestinal inflammation [33, 50] and other inflammatory diseases with no side effects. This may be because it is an essential nutrient [17, 51, 52]. Our data showed that n-3 FA was able to attenuate weight loss, damage of the mucosa, and apoptosis.

## Conclusion

Together, our results provide a new preventive treatment for mucositis and confirm the role of omega-3 fatty acid in the prevention of intestinal mucosal inflammation.

## Material and methods

### Animals, diet treatments, and mucositis induction

Male BALB/c mice between 6 and 8 weeks of age were purchased from Biotério Central of the Instituto de Ciências Biológicas da Universidade Federal de Minas Gerais (UFMG). Mice were housed at room temperature with water and food *ad libitum*. The UFMG Ethics Committee for Animal Experimentation (CETEA/UFMG) approved this study.

Mice were randomly divided into four groups as follows: 1. Control (CTL), fed standard chow diet; 2. CTL + n-3 FA, fed a diet with n-3 FA; 3. Mucositis (MUC), fed standard chow diet and underwent mucositis; and 4. MUC+ n-3 FA, fed a diet with n-3 FA and underwent mucositis. The CTL and MUC groups received the standard AIN-93G diet ad libitum. The CLT + n-3FA and MUC+ n-3FA groups were fed an experimental diet with added omega-3 fatty acid. Food consumption was calculated as the difference between the amount of offered chow and the residual chow. The individual amount of food ingested was calculated from the average of each cage. The weight of the mice was measured with a semi analytical balance.

The experimental diet was developed based on the AIN93G diet [53]. The AIN93G diet had 7 % lipids (soy oil). For the experimental diet, we used 3.5 % soy oil and 3.5 % fish oil. The total amount of lipids was divided into two parts, 50 % (35 g/kg of chow) soy oil and 50 % (35 g/kg of chow) fish oil. The new formulation is isocaloric compared to AIN93G.

The treatment with these diets was performed for one week prior to mucositis induction, and 24 h or 72 h after 5-FU injection. At the 8th day, mice in the MUC and MUC + n-3 FA groups received an intraperitoneal (IP) injection containing 300 mg/kg 5-FU, whereas the animals of the CTL and CTL + n-3FA groups received a saline IP injection. After 24 h (9th experimental day) or 72 h (11th experimental day), the mice were killed and assessed for intestinal permeability, bacterial translocation, intestinal histology and apoptosis assay.

### Diet lipid content analyzes

The fatty acid composition of the diet was determined by gas chromatography (model 6890 N chromatograph, Agilent Technologies) equipped with a fused silica capillary column (SP-2560, 100 m × 0.25 mm × 0.2 mm, Varian Inc.) and a flame ionization detector (FID). Before injection into the chromatograph, the lipid fractions in the feed solution were extracted into hexane-isopropanol as described by Hara and Radin (1978) [54], and the methyl esters were obtained with a basic sodium methoxide catalysis solution [55]. The fatty acids in the samples were identified by comparison to the retention times observed in standard commercial samples (Sigma Diagnostics) based on published articles [56, 57], and the quantification was performed by correcting the peaks to an area of 100 %. According to the lipid content analyses, only the diet made with fish oil had greater amounts of EPA and DHA fatty acids (Table 2).

**Table 2:** Lipid analysis of the standard diet and dibibet with n-3 FA

	Total FA	SFA	PUFA	n-3 FA	EPA	DHA	n-6 FA
CTL Chow	97,91 %	15,69 %	55,99 %	4,14 %	0,00 %	0,00 %	50,52 %
n-3FA Chow	78,66 %	8,68 %	54,40 %	24,61 %	15,28 %	7,00 %	26,54 %

The amount of fatty acid is expressed in % of the lipid content. The diet contains 7 % of the lipid

FA: fat acid; SFA: saturated fat acid; PUFA: polyunsaturated fat acid; EPA: eicosapentaenoic acid; DHA: docosahexaenoic acid

### Intestinal permeability determination

Intestinal permeability was determined by measuring the radioactivity diffusion in the blood after oral administration of diethylenetriaminepentaacetic acid (DTPA) labeled with 99 m-technetium (^99m^Tc) [58]. After 24 or 72 h of mucositis induction, all mice received 0.1 mL of a DTPA solution labeled with 18.5 mebequerel (MBq) of ^99m^Tc-DTPA by gavage. After four hours, all animals were anesthetized, and 300 μL of blood was collected and placed in the appropriate tubes for radioactivity determination (38). The data are expressed as % dose, using the following equation:

$$ \%\ \mathrm{Dose}=\left(\mathrm{cpm}\ \mathrm{of}\ \mathrm{blood}/\mathrm{cpm}\ \mathrm{of}\ \mathrm{administered}\ \mathrm{dose}\right)\times 100 $$

where cpm represents counts per minute.

### *E. coli* radio labeling and bacterial translocation determination

Bacterial translocation (BT) analysis was performed following a procedure described by Diniz et al. 1999 [59]. An *E. coli* ATCC10536 sample culture was grown overnight on tryptic casein agar (Difco) that was then transferred to a 10-mL sterile saline solution. The bacterial concentration was adjusted to 31 % of transmittance in a spectrophotometer at 580 nm, which corresponded to approximately 10^8^ CFU/mL. An aliquot of the bacterial suspension (2 mL) was incubated in tubes containing 1 mL of a stannous chloride solution (580 mM, pH 7.0) at 37 °C for 10 min. After incubation, 37.0–55.5 MBq of technetium-99 m (^99m^Tc), which was obtained by elution from a sterile 99Mo/99 m-Tc generator (IPEN/Brazil), was added, and the preparation was incubated at 37 °C for an additional 10 min. The tubes were then centrifuged at 3000 × g for 25 min. This procedure was repeated three times. After the last centrifugation, the radioactivity of the supernatant and precipitate was measured in a dose calibrator (CRC-25R Dose Calibrator, Capintec, Ramsey, USA), and the percentage of ^99m^Tc incorporated into the bacterial cells was determined using the following equation:

$$ =\left(\mathrm{cpm}\ \mathrm{of}\ \mathrm{precipitate}/\mathrm{cpm}\ \mathrm{of}\ \mathrm{precipitate}+\mathrm{cpm}\ \mathrm{of}\ \mathrm{supernatant}\right)\times 100 $$

where cpm represents counts per minute.

After 72 h of mucositis induction, 0.1 mL of a suspension containing 1.8 MBq ^99m^Tc-*E. coli* was administered by gavage to all of the animals. After four hours, the animals were anesthetized and the blood, mesenteric lymph nodes (MLN), livers and spleens were removed, weighed, and placed into the appropriate tubes for radioactivity determination. The samples were counted in a NaI (Tl) crystal counter (ANSR-Abott, Chicago, USA). The values are expressed as cpm/g or cpm/mL.

### Intestinal histology, morphometry, and apoptosis assay

#### Histology

After 24 or 72 h of mucositis induction, the ileum segments (distal 10 cm) were collected, fixed in methanol with 20 % dimethyl sulfoxide (DMSO) and stained with hematoxylin and eosin for villus height, crypt depth, lamina propria thickness, epithelium thickness, and villus thickness measurements. For each sample, ten intact villi pictures were taken and the parameters were measured.

### Morphology

For the morphological analysis, Image J software was used. The villus height was measured from the crypt apex adjacent to the villus apex. The crypt depth was measured from the submucosa to the villus apex. The villi thickness was measured as the largest horizontal villus thickness. The lamina propria thickness was measured as the major horizontal lamina propria thickness. The epithelium thickness was measured as the height of the enterocytes from the edge in contact with the lamina propria to the edge in contact with the intestinal lumen.

### Histochemistry

Cell apoptosis in the ileum was detected by a terminal deoxy nucleotidyl transferase-mediated dUTP nick-end labeling (TUNEL) assay using an in situ apoptosis detection kit (FragEL TM DNA Fragmentation Detection Kit, Colorimetric – Klenow Enzyme) according to the manufacturer’s instructions. The results are expressed as the presence or absence of TUNEL positive cells (brown color).

### Statistical analysis

The results are expressed as the means ± SD and were analyzed using GraphPad Prism version 5.0 (GraphPad Software, San Diego, CA). Multiple comparison analyses were performed using one-way ANOVA with Tukey post-hoc analysis. Statistical significance was set at *P* < 0.05.
